# Improving Accuracy of the Alpha–Beta Filter Algorithm Using an ANN-Based Learning Mechanism in Indoor Navigation System

**DOI:** 10.3390/s19183946

**Published:** 2019-09-12

**Authors:** Faisal Jamil, Do Hyeun Kim

**Affiliations:** Department of Computer Engineering, Jeju National University, Jejusi 63243, Korea; faisal@jejunu.ac.kr

**Keywords:** indoor navigation system, inertial measurement unit, artificial neural network, alpha–beta filter, motion trackina

## Abstract

The navigation system has been around for the last several years. Recently, the emergence of miniaturized sensors has made it easy to navigate the object in an indoor environment. These sensors give away a great deal of information about the user (location, posture, communication patterns, etc.), which helps in capturing the user’s context. Such information can be utilized to create smarter apps from which the user can benefit. A challenging new area that is receiving a lot of attention is Indoor Localization, whereas interest in location-based services is also rising. While numerous inertial measurement unit-based indoor localization techniques have been proposed, these techniques have many shortcomings related to accuracy and consistency. In this article, we present a novel solution for improving the accuracy of indoor navigation using a learning to perdition model. The design system tracks the location of the object in an indoor environment where the global positioning system and other satellites will not work properly. Moreover, in order to improve the accuracy of indoor navigation, we proposed a learning to prediction model-based artificial neural network to improve the prediction accuracy of the prediction algorithm. For experimental analysis, we use the next generation inertial measurement unit (IMU) in order to acquired sensing data. The next generation IMU is a compact IMU and data acquisition platform that combines onboard triple-axis sensors like accelerometers, gyroscopes, and magnetometers. Furthermore, we consider a scenario where the prediction algorithm is used to predict the actual sensor reading from the noisy sensor reading. Additionally, we have developed an artificial neural network-based learning module to tune the parameter of alpha and beta in the alpha–beta filter algorithm to minimize the amount of error in the current sensor readings. In order to evaluate the accuracy of the system, we carried out a number of experiments through which we observed that the alpha–beta filter with a learning module performed better than the traditional alpha–beta filter algorithm in terms of RMSE.

## 1. Introduction

The ability to navigate has always been of great importance when discovering new and unknown territories of the world. The evolution of various navigation techniques has helped us spread across the planet. Today, navigation remains an important part of our society. The technologies of today enable us to use the navigation in a whole new way than our ancestors could. Since smartphones were released on the market, a lot of location-based services have been developed. It is now possible to use navigation to find your way to a certain address or a point of interest, for example, the closest gasoline station or restaurant. All these functions are available because of Global positioning system (GPS) which has been integrated into those applications [1].

Nowadays, a GPS-based system is considered to be a well-known navigation system using satellites to calculate a receiver’s current location on earth. These systems take the user’s three-dimensional information (i.e., latitude, altitude, and longitude) [1,2,3]. The accuracy of GPS depends on the term of the line of sight; if the accuracy is good, then the system will easily locate the person or object within meters. Similarly, if the signal is weak, then the position of the object is unreliable, and the system is unable to get the exact position. Although GPS is considered to be very important for locating a target in an outdoor environment, it is not feasible for an indoor environment. In an indoor environment, there is always signal attenuation as compared to the outdoor environment because of weak signal or signal disturbed by impenetrable obstacles like a different object, concrete, and steel wall. These obstacles and hurdles continuously block signals coming from the satellites and, hence, it is difficult to locate an object’s precise location [3,4]. Therefore, in consideration of these problems, the GPS is not reliable for an indoor positioning system (IPS) [5].

The IPS is a system used to track and locate the position of the object or a person inside a building by using sensor data, magnetic field, acoustic signal, radio waves, and WLAN nodes. During the past decade, many significant research has been done in the field of indoor localization [6]. This has lead to the development of several IPSs using different technologies for both research and commercial purposes. Many GPS chips have been manufactured in order to get the location of the object in an indoor environment, but the output is not accurate as compared to an outdoor environment. The tracking of a person within an indoor environment is the common use case that many researchers have adopted in order to evaluate the system [7]. During the past several years, many devices have been made in order to get the object location through sensor data. These devices are called inertial measurement units (IMUs) [8].

Many IMUs have been developed in the past several years which use sensors like accelerometers, gyroscopes, and magnetometers in order to calculate object localization [9]. These sensor data are used to calculate the linear acceleration and angular rate of a moving body, respectively. There are many ways to calculate the distance of the moving object [10]. One of the popular ways is to take double integration of acceleration concerning time to get the distance of the moving object [11,12]. In the case of constant acceleration, motion can be characterized by the motion equation. The combined acceleration (*a*), time (*t*), displacement (*x*), and velocity (*v*) are described as Motion. The rate of change of displacement is defined as velocity, and the rate of change of velocity is called acceleration. The velocity calculation is shown in Equation (1), in which the velocity is obtained by integrating the constant acceleration. In Equations (2)–(4) the velocity is further integrate to get position of the object.

(1) $$v=\int adt=v_{o}+at$$

(2) y=∫vdt

(3) $$y=\int (v_{o}+at)dt$$

(4) $$y=y_{o}+v_{o}t+\frac{1}{2}at^{2}$$

Nevertheless, in these sensor values, there exists a dynamic noise of the accelerometer output, and it will increase with time by integrating the accelerometer [13]. Whenever a value is measured, there will be some error introduced by the transmission, as mentioned in Equation (5).

(5) measured_value=true_value+noise

In order to get the precise output, different kinds of filters have been used, e.g., Wiener filter, low-pass filter, Kalman filter, Gaussian filter, Butterworth filter, alpha–beta filter, and high-pass filter, etc. These filters are responsible for removing noise from the measured value [14]. Enabling a prediction algorithm to cope with dynamic data or changing location data is a challenging task [15]. In this article, we propose a general architecture to improve the performance of the prediction algorithm using the learning module. The learning module monitors the performance of the prediction algorithm continuously by receiving the output as feedback. After analyzing the noise strength in the measured value and the output of the prediction algorithm, the learning module updates the tunable parameter or swaps the trained model of the prediction algorithm to improve its performance in terms of prediction accuracy. For experimental analysis, we have used the alpha–beta filter as a prediction algorithm, and our learning module is based on an artificial neural network.

The rest of the paper is organized as follows: A detailed overview of related work is presented in Section 2. In Section 3, we present the proposed learning to prediction model and inertial tracking in indoor navigation with conceptual design and detailed description of the chosen case study. A detailed discussion of the implementation and experimental setup is presented in Section 4. Section 5 presents the results of the proposed system. Finally, we conclude the paper in Section 6.

## 2. Related Work

**Figure 1:**
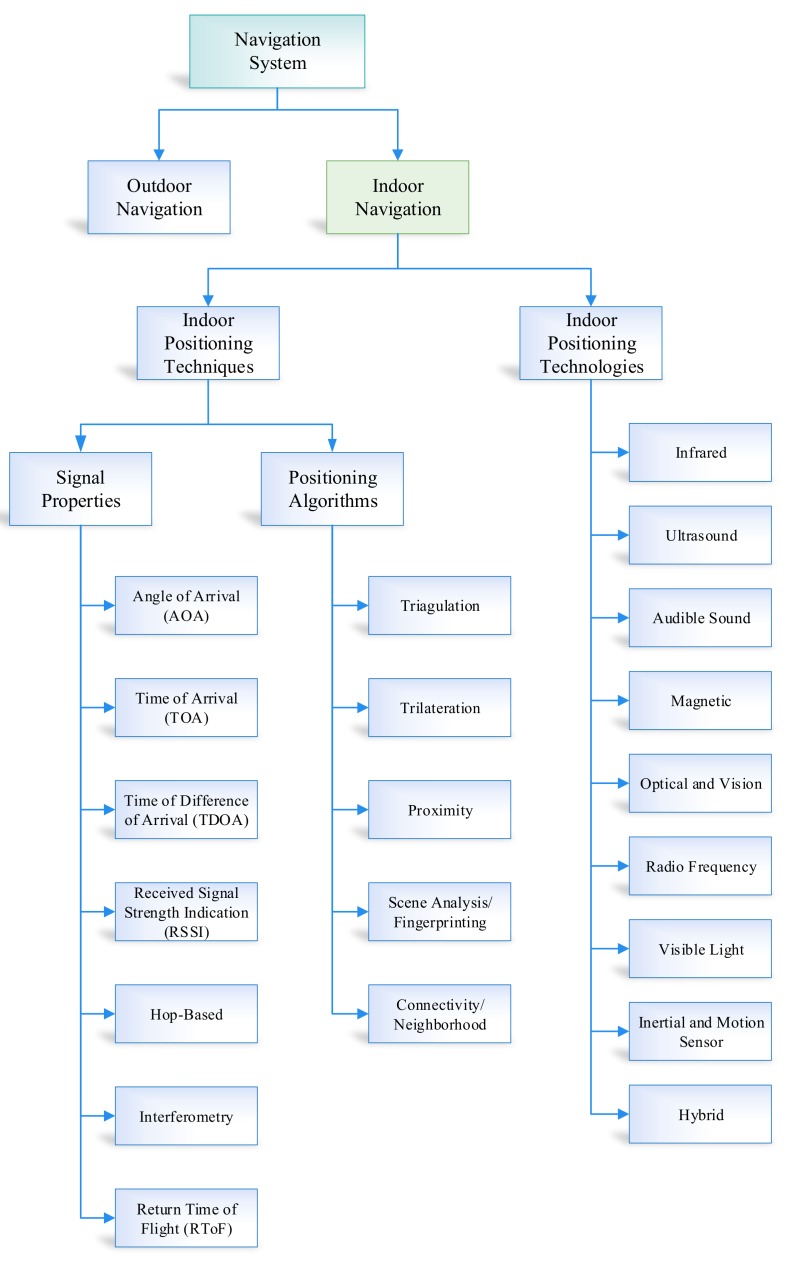
Taxonomy of indoor positioning algorithms.

Over the years, a lot of indoor positioning systems have been proposed to measure traveled distance. Navigation can be classified into two main categories, i.e., outdoor navigation and indoor navigation. The indoor navigation systems are further segregated into two main sub-categories, i.e., indoor positioning techniques and indoor positioning technologies. Furthermore, indoor techniques are further divided into two parts, i.e., signal properties and positioning algorithms. Location estimation and position algorithms are segregated into five categories, i.e., fingerprinting/Scene analysis, connectivity/neighborhood, triangulation, proximity, and trilateration [3]. Similarly, signal properties comprise seven types, i.e., Angle of Arrival (AoA), Time of arrival (ToA), Time of difference of arrival (TDoA), Received signal strength indication (RSSI), Hop-based, Interferometry, and Return time of flight (RToF). Finally, indoor positioning technologies are divided into ten categories, i.e., infrared, ultrasound, audible sound, magnetic, optical and vision, radio frequency, visible light, hybrid, inertial, and motion sensor. The overview of all these methods is presented in Figure 1. In this section, our main focus is to discuss the inertial and motion sensor in detail.

### 2.1. Inertial and Motion Sensor

In the inertial and motion sensor category, the distance of the object is calculated using the sensor’s value, i.e., gyroscope, magnetometer, and accelerometer. The magnetometer is used to determine the orientation relative to the earth’s magnetic field. The accelerometer is used to measure the acceleration of the object on a given axis. Similarly, the gyroscope is used to calculate the circular motion or angle of a moving object. From these sensors value, the double integration method over time yields the object’s velocity in the first step, and the second step calculates velocity to get a distance as illustrated in Equation (4). The Inertial and motion sensors are also used within the dead reckoning navigation. In dead reckoning navigation, the position estimation is calculated based on continuous tracking of the object using acceleration from the origin [3].

In [16], the authors implemented two algorithms which aim to measure distance. The distance is measured using the double integration of accelerometer. However, in double integration, the error rate is more than expected. In the second algorithm, the distance traveled is measured by counting the number of steps. The distance traveled by the steps is measured by calculating the angle between legs using the accelerometer and gyroscope. In order to remove the noise, the complementary filter is used in the proposed algorithm. The main advantage of this system is to reduce the circuit cost and increase the efficiency of the system.

A personal navigation system was presented in [17]. The developed system calculates the position of the pedestrian using the double integration method. The main aim of the system is to focus on three points; (i) real-time pedestrian position to get the accurate estimation, (ii) visualization of the position in 3D inside the building, and (iii) precise transition between the indoor and outdoor environments. The Kalman filter is also used to remove the sensing noise of the MTi/MT sensor in order to achieve accuracy. In [18], the authors proposed a new motion tracking system using two wearable inertial sensors. These inertial sensors are placed on upper limb joints near the wrist and elbow. An MT9B sensor is used which contains a 3-axis accelerometer, gyroscope, and magnetometer sensor in order to detect the motion of the human wrist, elbow and shoulder. In order to estimate the shoulder position, a Lagrangian-based optimization method was then adopted, integrating the translation and rotation components of the wearable inertial sensors.

The Kinematic-based model is designed to control the robotic arm using a dynamic state–space method in order to estimate the angle of the human shoulder using two wearable inertial sensors. In order to eliminate the noise, the Kalman filter has been used to implement the nonlinear state–space inertial tracker. The performance of the system is calculated in terms of RMS angle error, which is less than $8^{\circ }$ for both shoulders and arms. Moreover, the average correlation is r≥0.95 for all movement tasks [19]. In [20], the authors presented an inertial tracking for mobile augmented reality. Real-time tracking is computed using an accelerometer, gyroscope, and silicon micro-machined. Six DoF are used to visualize the real-time movement and are capable of visualizing the movement in an indoor and outdoor environment.

Authors in [21] proposed a new method that detects the period of eating using a watch-like configuration. The sensor monitors the movement of wrist all day and detects whether the person is eating or not. The main aim of this study is to monitor the daily activity of a person in terms of energy intake.

#### 2.1.1. Dead Reckonina

The inertial and motion sensors are used within the so-called dead reckoning navigation. Dead Reckoning (DR) is also known as pedestrian dead reckoning (PDR) and is a mechanism for estimating the user’s current position using the previously known position with respect to time. DR is an alternative of radio navigation like the GPS, e.g., in case of bad weather due to signal attenuation, the GPS fails to work properly [1,22]. DR can give accurate position information, but it will give an error for a long periods of time [23]. In order to improve the accuracy of DR, the new hybrid solution is presented by the author which is more reliable than the existing solutions [24]. DR is also used with inertial navigation systems (INSs) such as a PDR in order to provide an accurate position estimation [25]. Similarly, DR is also embedded in micro-electromechanical systems (MEMS) to develop miniaturized electromechanical navigation device systems which are more reliable, accurate and have a low cost [26,27].

INS uses an inertial sensor to estimate the acceleration, position, velocity, and orientation of the object in motion with the involvement of external reference points [1,28]. This estimation of position, velocity, acceleration, and orientation is possible using DR integrated with inertial sensors, i.e., accelerometer, gyroscope, and magnetometer in order to attain an accurate estimation [29]. The common algorithm used in pedestrian navigation is Extended Kalman Filter (EKF), Particle Filter (PF), Kalman Filter (KF) integrated with INS to predict the position in indoor environment [23,25,30].

The authors in [31] presented the feasibility of using only the magnetic field for indoor positioning. The advantage of only using the magnetic field for position estimation in indoor environments is that no infrastructure is required to be deployed for the designed system which makes this approach cost-effective. Moreover, the performance of the system is directly proportional to the number of fingerprints. The magnetic field intensity data comprised of three groups, i.e., intensities in X, Y, Z direction. Furthermore, the magnetic field is unknown even with the integration of acceleration, i.e., horizontal intensity and vertical intensity.

In [32], the authors presented a VMag an infrastructure-free indoor positioning system fusion with magnetic and visual sensor. The proposed system is based on a novel approach for estimating the position in an indoor environment without relying on pre-deployed infrastructure assistance. The localization can be easily done by a user holding a smartphone. The presented system is designed using a particle filtering framework integrated with a neural network which improved the accuracy of the localization in an indoor environment. A number of experiments are carried out for different indoor settings, e.g., a laboratory, a garage, a canteen, and an office building.

Based on the comprehensive analysis of the state-of-the-art approaches in the field, limitations of available indoor techniques are described in Table 1, Table 2 and Table 3.

Previously, there have been a lot of research proposed for increasing the performance and accuracy of the motion tracking and navigation systems using different algorithms, except for the alpha–beta filter algorithm. Nevertheless, none of these systems address the tuning of prediction algorithm with ANN. To the best knowledge of the authors, there has been no functional, positioning system for indoor navigation systems based on a learning to prediction model built so far.

## 3. System Architecture of Proposed Indoor Navigation

### 3.1. Scenario of Inertial Tracking in Indoor Navigation

**Figure 2:**
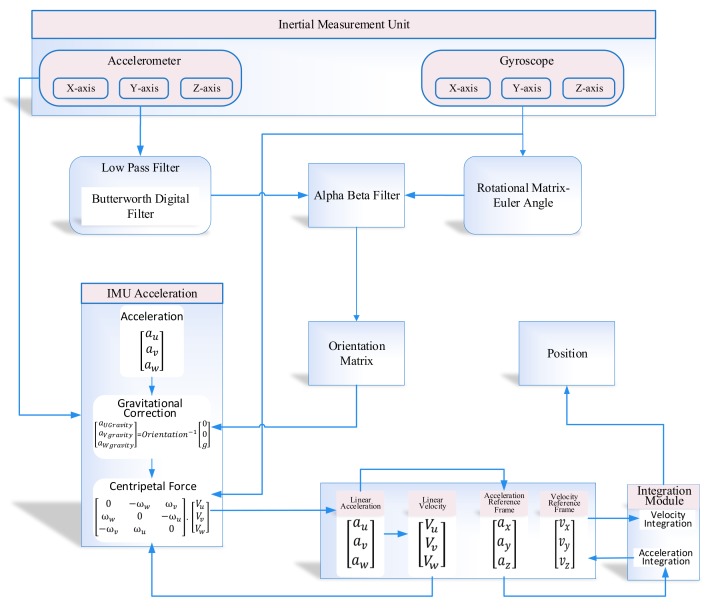
Inertial tracking scenario for proposed indoor navigation system.

The three-axis output from IMU in the form of linear acceleration and angular velocity is combined in the form of the non-linear matrix equation. The non-linear matrix has the information of both the orientation and position of the object in an indoor environment. The orientation of the object is calculated in two ways, i.e., orientation estimation from gyroscope output and orientation estimation from accelerometer output. In the case of the gyroscope, the absolute orientation cannot be calculated directly because of the drift associated with gyroscope readings. However, in the acceleration case, the orientation estimation is calculated using short-term and long-term stability. The short-term orientation stability is inaccurate as compared to long-term orientation stability because of the presence of ferromagnetic material. In other cases, the orientation matrix accuracy is disturbed due to rotational and linear acceleration. In order to fix the problem of orientation inaccuracy, we combined the advantages of long-term stability of accelerometer and short-term precision of gyroscope via an alpha–beta filter, as shown in Figure 2.

#### 3.1.1. Orientation Estimation from Gyroscope in Indoor Navigation

The orientation estimation from gyroscope output in indoor navigation is measured using the Euler angles (ψ, ϕ, and θ). The Euler angle is used to describe the orientation of an object in 3-dimensional Euclidean space. Furthermore, we also keep track of the order of the rotation in every time step, which is as follow.

(6) $$\Delta \theta \left(P+1\right)=\Delta t\frac{\theta (P+1)+\theta (P)}{2}$$

(7) $$\Delta \phi \left(P+1\right)=\Delta t\frac{\phi (P+1)+\phi (P)}{2}$$

(8) $$\Delta \psi \left(P+1\right)=\Delta t\frac{\alpha (P+1)+\alpha (P)}{2}$$

We use the trapezium rule as numerical integration method in above three equation and these Equations (6), (7), and (8) represents the Yaw, Pitch and Roll. Where ΔP is the time index, Δθ, Δψ, and Δϕ denotes the incremental angle around the W-axis, U-axis, and V-axis.

The rotation matrix mention in Figure 2 is denoted as *R* in (9), (10), and (11) around each particular axis.

(9) $$R\left(W,\theta ,P+1\right)=\left(\begin{array}{ccc} cos\Delta \theta (P+1) & -sin\Delta \theta (P+1) & 0\\ sin\Delta \theta (P+1) & cos\Delta \theta (P+1) & 0\\ 0 & 0 & 1 \end{array}\right)$$

(10) $$R\left(V,\phi ,P+1\right)=\left(\begin{array}{ccc} cos\Delta \phi (P+1) & 0 & sin\Delta \phi (P+1)\\ 0 & 1 & 0\\ -sin\Delta \phi (P+1) & 0 & cos\Delta \phi (P+1) \end{array}\right)$$

(11) $$R\left(U,\psi ,P+1\right)=\left(\begin{array}{ccc} 1 & 0 & 0\\ 0 & cos\Delta \psi (P+1) & -sin\Delta \psi (P+1)\\ 0 & sin\Delta \psi (P+1) & cos\Delta \psi (P+1) \end{array}\right)$$

In (9), (10), and (11) are combined in form of general rotation matrix represent in (11).

(12) Rotation(P+1)=R(W,θ,P+1).R(V,ϕ,P+1).R(U,ψ,P+1)

Finally, the orientation matrix is defined in (13).

(13) Orientation(K+1)=Rotation(K+1).Orientation(K)

The orientation matrix contains information related to the orientation of IMU. Therefore, in order to get the orientation matrix, we process the coordinates of any vector from the IMU-Fixed Frame to the Earth-Fixed Frame.

#### 3.1.2. Orientation Estimation from Accelerometer in Indoor Navigation

In the proposed Indoor navigation system, the orientation matrix is a 3 × 3 matrix that transforms vectors linked with IMU-Fixed Frame into an Earth-Fixed Frame. The impact of the transformation of the vector in the proposed method is to transform orientation matrix coordinates from IMU-Fixed Frame into Earth-Fixed Frame, and similarly, the inverse transforms from Earth-Fixed Frame to IMU-Fixed Frame. The inverse of the orientation matrix is defined in (14).

(14) $$orientation^{-1}=\left(\begin{array}{ccc} a_{11} & a_{12} & a_{13}\\ a_{21} & a_{22} & a_{23}\\ a_{31} & a_{32} & a_{33} \end{array}\right)$$

We also calculate the acceleration magnitude, which is denoted by a, since the vector is referenced to Earth-Fixed Frame, which is parallel to the z-axis. Therefore, the accelartion vector coordinates in the Earth-Fixed Frame are (0 0 g) and, ($a_{ua}$$a_{va}$$a_{wa}$) is the acceleration field vector directly measured from the IMU output. In this case, the accelartion vector coordinates concerning Earth-Fixed Frame is represented in (15).

(15) $$\left(\begin{array}{c} a_{ua}\\ a_{va}\\ a_{wa} \end{array}\right)=\left(\begin{array}{ccc} a_{11} & a_{12} & a_{13}\\ a_{21} & a_{22} & a_{23}\\ a_{31} & a_{32} & a_{33} \end{array}\right)\cdot \left(\begin{array}{c} 0\\ 0\\ g \end{array}\right)$$

where the acceleration vector to Earth Fixed-Frame is also represented in (15), where g denotes the magnitude of acceleration. The inverse of the orientation matrix is mentioned in (15) and (16):

(16) $$\left(\begin{array}{c} a_{13}\\ a_{23}\\ a_{33} \end{array}\right)=\frac{1}{a}\cdot \left(\begin{array}{c} a_{ua}\\ a_{va}\\ a_{wa} \end{array}\right)$$

Lastly, across a product from the third and first column, we compute the second column inverse of the orientation matrix. The proposed system is comprised of three main components, i.e., orientation matrix, linear acceleration, and position tracking. Therefore, the equation for inertial tracking is represented in (17).

(17) $$X\left(P+1\right)=\alpha \cdot \left[Rotation\left(P+1\right)\cdot X\left(P\right)\right]+\beta \cdot \left[Orientation\left(P+1\right)\cdot \frac{\left(P+1)+(P\right)}{\left(P\right)}\right]$$

Equation (17) used trapezium rule as the numerical integration method where.

(18) $$X=\left(\begin{array}{c} x\\ y\\ z\\ \dot{x}\\ \dot{y}\\ \dot{z} \end{array}\right)$$

Equation (18) represents the position and velocity of the object in a proposed indoor environment where *x*, *y*, *z* are the 3-axis position and $\dot{x}$, $\dot{y}$, $\dot{z}$ are the velocity of objects.

(19) $$\alpha =\left(\begin{array}{cccccc} 1 & 0 & 0 & \Delta t & 0 & 0\\ 0 & 1 & 0 & 0 & \Delta t & 0\\ 0 & 0 & 1 & 0 & 0 & \Delta t\\ 0 & 0 & 0 & 1 & 0 & 0\\ 0 & 0 & 0 & 0 & 1 & 0\\ 0 & 0 & 0 & 0 & 0 & 1 \end{array}\right)=\left(\begin{array}{cc} I_{3x3} & \Delta t.I_{3x3}\\ o_{3x3} & I_{3x3} \end{array}\right)$$

(20) $$A_{acc}=\left(\begin{array}{c} a_{u}\\ a_{v}\\ a_{w} \end{array}\right)$$

(21) $$L_{vec}=\left(\begin{array}{c} V_{U}\\ V_{V}\\ V_{W} \end{array}\right)$$

Equation (20), $A_{acc}$ represent the acceleration of the object in proposed indoor navigation and similarly (21) represents the linear velocity vector denoted as $L_{vec}$.

(22) $$\beta =\left(\begin{array}{c} \frac{\Delta t^{2}}{2}\cdot I_{3x3}\\ \Delta t\cdot I_{3x3} \end{array}\right)$$

(23) $$Rotation\left(P+1\right)=\left(\begin{array}{cc} I_{3x3} & O_{3x3}\\ O_{3x3} & Rotation(P+1) \end{array}\right)$$

In Equation (23) the identity is represented by 1, and Null matrices are denoted by 0.

In the proposed inertial tracking scenario, we integrate linear acceleration to get the linear velocity in order to get the position in the proposed indoor system. However, to get an accurate position, we have to remove both the centripetal force from the data used in the linear acceleration. In Figure 2, IMU acceleration module has three-component, i.e., linear acceleration and centripetal acceleration. Therefore the IMU acceleration in Fixed-Frame is calculated using (24).

The rotation of the object can be measured using two ways, e.g., (i) self-rotation of the body and (ii) body rotation around a point in space. However, in the proposed indoor navigation system, we only consider body rotation around a point in space; therefore, we calculate the centripetal force using the linear velocity and angular velocity cross product. The result of this cross product is the linear velocity concerning IMU Fixed-Frame with the axis(*U*, *V*, *W*) which is represented in (26).

(24) $$\left(\begin{array}{c} a_{Ua}\\ a_{Va}\\ a_{Wa} \end{array}\right)(t+\Delta t)={Rotation}^{-1}(t+\Delta t)\left(\begin{array}{c} a_{Ua}\\ a_{Va}\\ a_{Wa} \end{array}\right)\left(t\right)$$

Similarly, using (24) we represent the orientation matrix in (25) as follow:

(25) $$\left(\begin{array}{c} a_{Ua}\\ a_{Va}\\ a_{Wa} \end{array}\right)\left(t\right)={Orientation}^{-1}\left(t\right)\left(\begin{array}{c} 0\\ 0\\ g \end{array}\right)$$

Finally, gravitational acceleration with respect to IMU Fixed-Frame is represented in (26).

(26) $$\left(\begin{array}{c} a_{Ua}\\ a_{Va}\\ a_{Wa} \end{array}\right)$$

(27) $$\left[\begin{array}{c} a_{ucentripetal}\\ a_{vcentripetal}\\ a_{wcentripetal} \end{array}\right]=\left[\begin{array}{ccc} 0 & -\omega_{w} & \omega_{v}\\ \omega_{w} & 0 & -\omega_{u}\\ -\omega_{v} & \omega_{u} & 0 \end{array}\right]\cdot \left[\begin{array}{c} V_{U}\\ V_{V}\\ V_{W} \end{array}\right]$$

### 3.2. Proposed System Architecture of Learning to Prediction Scheme

**Figure 3:**
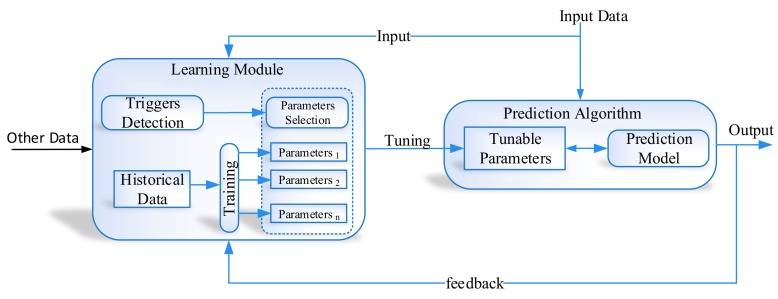
Conceptual view of learning to prediction model.

The proposed learning to prediction scheme is comprised of the prediction algorithm and learning module. Traditionally, we trained prediction algorithms using historical data, so that they can learn the hidden pattern and relationship among input and output parameters. Subsequently, trained models are used to predict the output for any given input data. The prediction algorithm will perform well when input data and the application scenario remain the same as the training data conditions. However, the existing prediction algorithm does not allow adaptation of the trained model with changing and dynamic input conditions. To overcome this limitation, we propose the learning to prediction model, as shown in Figure 3. In order to improve the accuracy of the prediction algorithm, we tune the prediction algorithm using a learning module. In the proposed system, the performance of the prediction algorithm is contentiously monitored by the learning algorithm by receiving its output as feedback. Moreover, the performance of the prediction algorithm also depends on external parameters, which are considered by the learning module. After investigating the output and the current external factors of the prediction algorithm, the learning module may update the tunable parameters of the prediction algorithm or completely replace the trained model in a prediction algorithm to improve its performance in terms of prediction accuracy when environmental triggers are observed.

**Figure 4:**
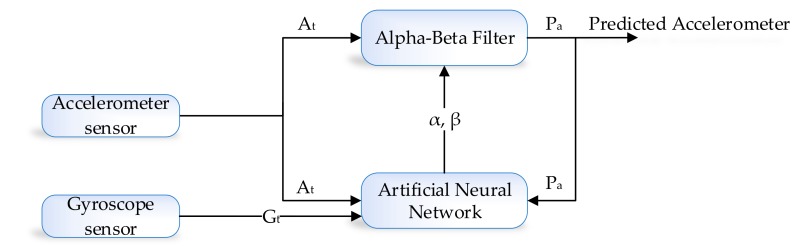
Accelerometer and gyroscope prediction using ANN-based learning module.

The development environment of the proposed system is categorized into two parts, i.e., the learning module and prediction algorithm. The prediction algorithm is based on the alpha–beta filter, and for the learning module, we used the artificial neural network (ANN). An alpha–beta filter is a simplified form of the observer for data smoothing, control, and estimation application. An alpha–beta filter is a lightweight algorithm that does not require historical data, but only previous state information to make an intelligent prediction about the actual state of the system. In the proposed method, the alpha–beta filter is used to predict the actual IMU sensor value, i.e., accelerometer and gyroscope from the noisy IMU sensor value. Noise in accelerometer sensor readings is due to the gyro bias. However, in the case of the learning module, we choose the ANN algorithm which takes three input, i.e., acceleration, gyroscope and previously predicted value (feedback) as shown in Figure 4.

**Figure 5:**
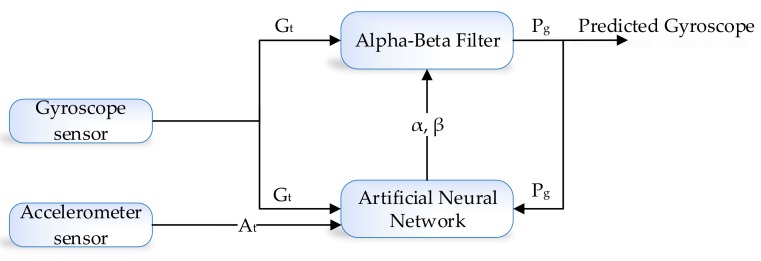
Gyroscope prediction using ANN-based learning module.

Similarly, in the case of gyroscope prediction, the noise in gyroscope is due to the influence of accelerometer value. For the learning module, we take three input, i.e., gyroscope, acceleration, and previously predicted value(feedback) as shown in Figure 5.

The alpha–beta filter algorithm gets readings from the IMU sensor at time *t* i.e., $A_{t}$ and $G_{t}$, and will predict accelerometer and gyroscope $P_{a}$ and $P_{a}$ data by removing noise. The performance of the alpha–beta filter algorithm’s is mainly controlled through a tunable parameter known as α and β that is calculated at each iteration. The residual (*r*) in the sensors reading is computed by the learning module so that α and β can be updated intelligently. In the next subsection, we will explain the detail of the alpha–beta filter before going into detailed architecture.

### 3.3. Alpha-Beta Filter Algorithm

**Figure 6:**
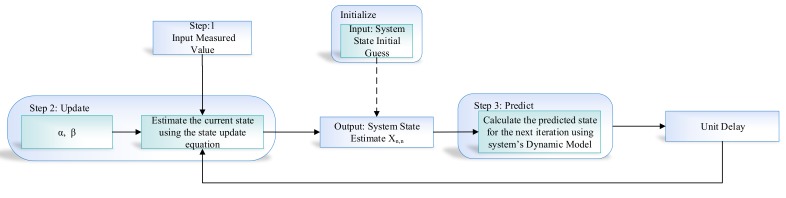
Working of the alpha–beta filter algorithm.

An alpha–beta filter is a simplified form of an observer for data smoothing, estimation, and control application. It consists of two internal states in which the first state is obtained by integrating the value of the second state over time. The output value of the system corresponds to the observation of the first state and disturbance. The α and β gain is the most important parameter of the alpha–beta filter’s design, which is the main key point behind the performance of the algorithm. An alpha–beta algorithm updates the value of gain depending on the situation to control weights given to the system’s own predicted state or sensor readings. The detailed working with all the components of the alpha–beta filter are present in Figure 6.

The noise in the sensor value depends on environmental factors, which can seriously affect sensor readings in that environment. In this study, we consider an IMU sensor reading (i.e., accelerometer and gyroscope) having noise, and let us assume $P_{a}$ and $P_{a}$ and an accelerometer and gyroscope at time t. The alpha–beta filter algorithm includes the process model that can make an internal prediction about the system state, i.e., estimated accelerometer and estimated gyroscope, and then, it is compared with the current sensor reading to decide predicted accelerometer and predicted gyroscope $T_{t+1}$ at time t+1. The αβ filter is commonly used as an efficient tracking filter. It may be viewed as the steady-state of the second-order Kalman filter. Next, we briefly explain the step-by-step working of alpha–beta filter algorithm, that is, how it removes the noise from the IMU sensor data.

(28) $${\hat{X}}_{K|K-1}=F{\hat{X}}_{K-1|K-1}=\left[\begin{array}{cc} 1 & T\\ 0 & 1 \end{array}\right]{\hat{X}}_{K}-1|K-1$$

In (28) ${\hat{X}}_{K|K}$ and ${\hat{X}}_{K|K-1}$ represents the estimated and predicted vector state at time KT.

(29) $${\hat{X}}_{K|K}=F{\hat{X}}_{K|K-1}+K_{V_{K}}={\hat{X}}_{K|K-1}+\left[\begin{array}{c} \alpha \\ \beta \\ \bar{T} \end{array}\right]V_{K}$$

where $\bar{T}$ represent the sampling period, in (29), the steady-state Kalman filter gain is represented by *K*, where $V_{K}$ is the innovation process which is further defined in (30).

(30) $$V_{K}=Z_{K}-{\hat{Z}}_{K}$$

In Equation (30), $Z_{K}$ is the *K*th measurement vector and $Z_{K}$ represents its predicted value, as mentioned by (31).

(31) $${\hat{Z}}_{K}=H{\hat{X}}_{K|K-1}$$

where *H* represent the measurement matrix, as mentioned in (32)

(32) $$H=\left[\begin{array}{cc} 0 & 1 \end{array}\right]$$

Finally the α, β coefficients is calculated as follow:

(33) $$\beta =\frac{\alpha^{2}}{2-\alpha }$$

However, the λ is represented as tracking index. The λ function is the variance and the measurement noise, receptively.

(34) $$\lambda =\frac{\rho_{v}}{\rho_{w}}T^{2}$$

The $\rho_{v}$ in (34) is represented as standard deviations of the system noise and $\rho_{w}$ is the measurement noise respectively. The ideal value of α and β is calculated using the (35)

(35) $$\alpha =-\frac{1}{8}\left(\lambda^{2}+8\lambda -\left(\lambda +4\right)\sqrt{\lambda^{2}+8\lambda }\right)$$

(36) $$\beta =2\left(2-\alpha \right)-4\sqrt{1-\alpha }$$

### 3.4. ANN-Based Learning to Prediction for the Alpha–Beta Filter

**Figure 7:**
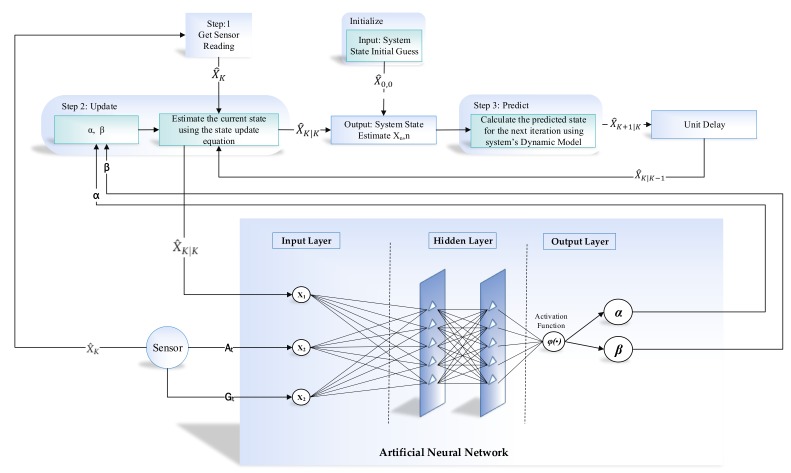
Detailed diagram of accelerometer and gyroscope prediction using the alpha–beta filter with learning module.

ANN algorithms are general-purpose learning algorithms and are actively used in solving a wide range of problems, including regression, classification, clustering, pattern recognition, forecasting, and time-series data processing. In the proposed indoor navigation system, the alpha–beta filter algorithm [33] is controlled using the ANN-based learning module. Figure 6 illustrates the flow diagram of the operation of the alpha–beta filter, which works fine with the optimal value of α and β. In the proposed system, we predict the actual value of accelerometer and gyroscope from the noisy sensor reading. The conventional alpha–beta filter fails to predict the actual sensor value under dynamic conditions. In Figure 7, we proposed the detailed learning to prediction model, which is based on an artificial neural network algorithm taking three inputs, i.e., current sensor value (i.e., accelerometer and gyroscope) and previously predicted sensor values. The output of the ANN algorithm is the optimal value of α and β. The updated value of α and β is passed to the filter algorithm to tune its prediction accuracy by adjusting the αβ value. The number of neurons defineed in the hidden layer are ten; we defined ten hidden layers because it was the best compromise between accuracy and efficiency; the number of neurons in the output layer is two. The tang-sigmoid function has been used as an activation function. The ANN provides α, and β values as output to the filter algorithm used in (29).

The proposed learning to prediction model enables the alpha–beta filter to estimate the actual accelerometer and gyroscope accurately from the noisy sensor reading with a dynamic error rate.

## 4. Implementation for ANN-Based Learning Mechanism in Indoor Navigation

### 4.1. Development Environment

The development environment of the proposed system is categorized into two parts, as illustrated in Figure 2 and Figure 3, i.e., inertial tracking in indoor navigation and the learning to prediction model. For inertial tracking in the indoor navigation system, we have used NGIMU [34], which is a data acquisition platform that combines on-board sensors and data processing algorithms. The NGIMU contains a triple-axis accelerometer and gyroscope sensor [34]. The detailed characteristics of NGIMU are described in Table 4.

The NGIMU sensor in the proposed system is used to collect data for inertial tracking of an object in an indoor environment. Therefore, the data were taken from two different locations while making the object navigate in each respective location. First, the data were taken from the movement of an object from the main corridor to room No. D243 in the Engineering building-4. Afterwards, the same pattern was followed while the object was moving in the conference room in Ocen Science building of Jeju National University, South Korea. The time duration for collecting one sample is approximately 60 s in which the first 10 s remained stationary so that the algorithm could converge on a stable state. The detailed development environment of inertial tracking in the indoor navigation system is summarized in Table 5.

Similarly, in the case of the learning to prediction model, we have used ANN for learning module and alpha–beta filter as a prediction algorithm. The Tool and technologies for implementing learning to prediction model mentioned in Table 6. All the implementation and experimental work of this study were carried out on Window 10 with an Intel Core i5-8500@3.00GHz processor and 8 GB memory. Moreover, we use MATLAB R2018a as a development environment for both inertial tracking and learning to the prediction model. In order to make the system friendly and efficient, we use the third party tool, i.e., NGIMU application programming interface (API) to extract data from the NGIMU sensor.

### 4.2. Implementation

**Figure 8:**
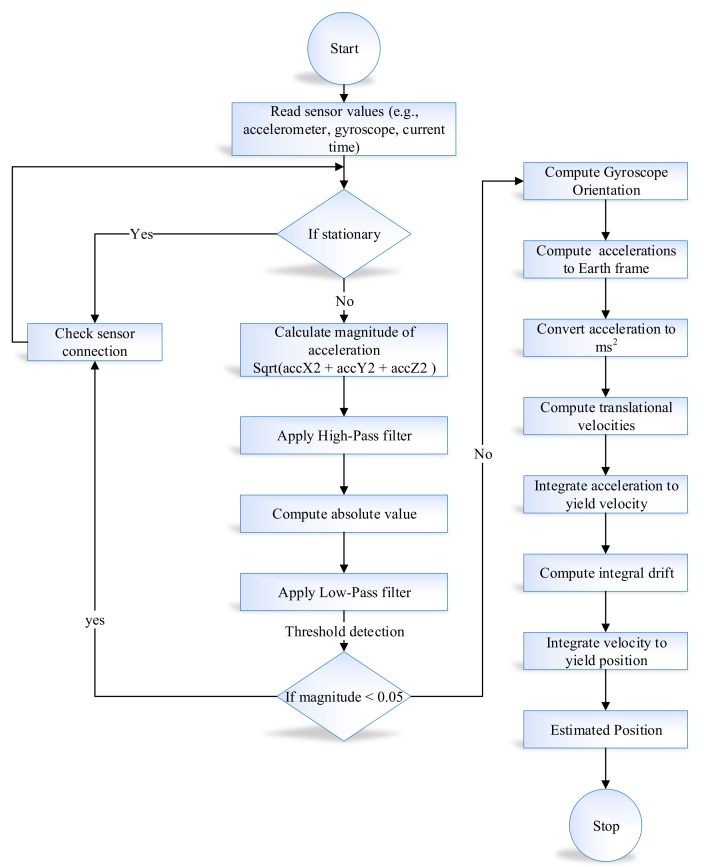
Flow chart of inertial tracking in indoor navigation.

We implemented the proposed system for the evaluation of the alpha–beta filter algorithm with the learning module in MATLAB R2018a. The implementation of the proposed system is comprised of two modules, i.e., Learning to prediction module and inertial tracking for indoor navigation. In inertial tracking for indoor navigation, the experiments were performed on a real dataset containing an accelerometer and gyroscope data. The data were taken from two different locations while making the object navigate in each respective location. First, the data were taken from the movement of object from the main corridor to room No. D243 in engineering building-4. Afterwards, the same pattern was followed while the object was moving in the conference room in Ocen Science building-5 of Jeju National University. The time duration of collecting a sample is 60 s. We loaded the data into the proposed application data structure from the Excel file. The data contain three inputs (i.e., accelerometer, gyroscope, and previously predicted value of sensor). First, we retrieved sensor data along with current time. After that, we calculated the magnitude of the acceleration and applied a low pass Butterworth digital filter to remove noise from the sensor data. Similarly, in the case of a gyroscope, we also computed magnitude first and then the rotational Matrix–Euler angle. The absolute value was then passed to the alpha–beta filter for further signal smoothing. After the orientation matrix was calculated, it was used to perform a rotation. Finally, when all bias from the data was removed, we used the traditional position estimation method, which is a double integration method. In a double integration method, first, we calculate the linear velocity through the integration of linear acceleration, then we calculate the position by taking the integration of linear velocity. The detailed flow chart of inertial tracking for indoor navigation is described in Figure 8.

In Learning to prediction module, first, we computed the root mean squared error (RMSE) for the sensor readings by comparing its values with the original sensor data, i.e., accelerometer and gyroscope data. The RMSE for the sensor reading is very high, i.e., 5.32.

Next, we used the alpha–beta filter algorithm to predict actual accelerometer and gyroscope readings from the noisy sensor reading. The implementation interface provides manual tuning of the alpha–beta filter internal parameter, i.e., time, current system state, a current derivate of the system state. Experiments were conducted with different values of input parameters, and the corresponding results were collected. The RMSE for the predicted accelerometer and gyroscope value using Alpha-Beta filter with α=0.5 and β=0.1 was 2.59, which was much better than RMSE of the sensor reading, i.e., 53.32% reduction of the error. However, it still needs improvement. We have to use MATLAB for the implementation of the ANN-based learning module to predict and tune the α and β to improve the prediction accuracy of the alpha–beta filter algorithm. The ANN algorithm has three neurons in the input layer for current sensor value (i.e., accelerometer and gyroscope) and predicted sensor values and two neurons in the output layer for prediction the error in sensor readings. Input and output data were normalized using the Equation mentioned in (37).

(37) $$\tilde{n_{i}}=\frac{n_{i}-n_{min}}{n_{max}-n_{min}}$$

The $\tilde{n_{i}}$ is the normalized value for the *i*th data point of the input and the output parameters, i.e., current sensor reading (i.e., accelerometer and gyroscope) and predicted sensor values, and the predicted α and β value. The minimum and maximum values in the available dataset is represented by $n_{min}$ and $n_{max}$ respectively. Usually the ANN network is trained with the normalized data, therefore, we need to de-normalize the output of the neural network to get the corresponding predicted error using (38).

(38) $$error_{i}=\tilde{error_{i}}x\left(error_{max}-error_{min}\right)+error_{min}$$

**Figure 9:**
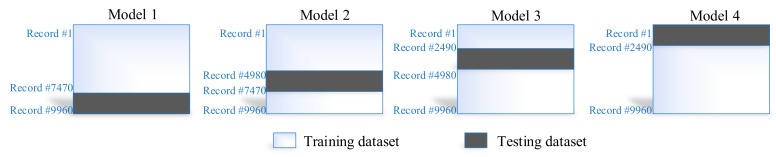
Training and testing dataset in four-fold cross-validation.

In the proposed system, we considered different configurations (e.g., the activation functions, changing neurons number in the hidden layer, learning rates) for the training of the ANN algorithm. We carried out multiple experiments for the training of every individual configuration of ANN; average results are reported to factor out the stochastic element in ANN network weights’ initialization. Moreover, we have used the four-fold cross-validation technique to evade the bias in the training process. The four-fold cross-validation is used for every individual configuration in the proposed system. In this case, we divided our dataset into four subsets of equal size (i.e., 2490 instances in each subset). The training and testing dataset used for each model in our four-fold cross-validation process is illustrated in Figure 9.

According to this technique, 25% of the dataset is used for testing, and the remaining 75% is used for training the ANN algorithm. The ANN training algorithm was based on the Levenberg–Marquardt algorithm, which is considered to be the best and fastest method for moderately sized neural networks. The maximum number of epochs used to train the ANN was 100. The selected configuration for ANN along with the corresponding prediction accuracy in terms of RMSE for training and the testing dataset are summarized in Table 7.

Table 7 shows that the ANN prediction accuracy in terms of RMSE is affected by changing the learning rate or changing neuron number in the hidden layer. Nevertheless, a significant difference in prediction accuracy can be seen for each model in the four-fold cross-validation process. In the ANN algorithm, the sigmoid activation function is commonly used for prediction accuracy. In the proposed indoor navigation system, the sigmoid activation function performs better, and significant improvement can be observed as compared to the linear activation function. The highlighted column shows the best case achieved for the ANN algorithm using a sigmoid activation function with learningrate=0.2 and neuron=10. The best case configuration is further used for tuning the performance of the alpha–beta algorithm.

## 5. Results and Discussions

We have used an open-source NGIMU API for collecting real-time accelerometer and gyroscope data to calculate the inertial tracking in an indoor environment. Furthermore, in order to analyze the performance of the proposed system, we compared the proposed learning to prediction model with conventional alpha–beta filter to observe the improvement in the prediction accuracy of the alpha–beta filter algorithm results. For the traditional filter, the result was collected with varying the value of α and β. Hence, the proposed system is comprised of two modules (i.e., the inertial tracking in indoor navigation module and learning to prediction module); therefore, in this section, we first demonstrate and discuss the inertial tracking in indoor navigation module results and then learning to prediction.

**Figure 10:**
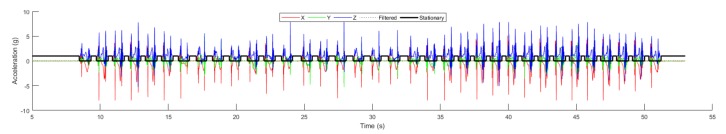
Acceleration.

The inertial tracking required sensor data (i.e., accelerometer and gyroscope) which is taken using NGIMU in Jeju National University, South Korea. Figure 10 investigated the accelerometer data which is collected form NGIMU sensor. The 3-axis accelerometer data with respect to time are shown along with the filtered and stationary data. The stationary data depict the accelerometer magnitude less than 0.05 to check the state object. Similarly, we use the Butterworth filter to filter the accelerometer data using the specified cut off frequency.

**Figure 11:**
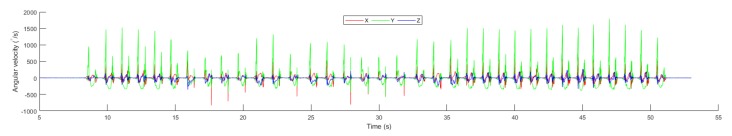
Angular velocity.

Figure 11 shows the angular velocity of the object in an indoor environment calculated using gyroscope data. The angular velocity is used as the object moves through an angle. It is the change in perspective of a moving object divided by time. The angular velocity is calculated using the following formula.

(39) $$\omega =\frac{\left(\theta_{f}-\theta_{i}\right)}{t}$$

In Equation (39), ω represent the angular velocity, $\theta_{f}$ is final angle of the object, $\theta_{i}$ is the initial angle of the object, *t* represent the time, and Δθ is the change of angle.

**Figure 12:**
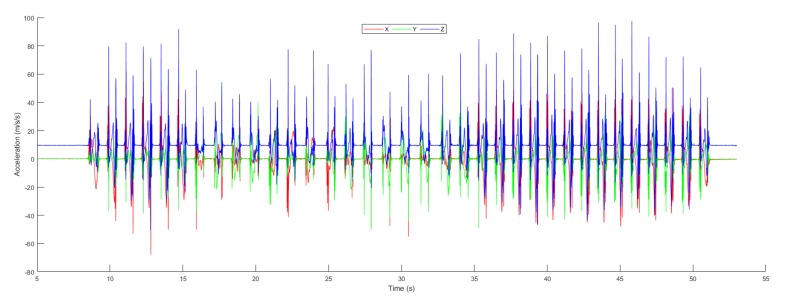
Acceleration $m/s^{2}$.

The 3-axis acceleration of the object in an indoor environment is illustrated in Figure 12. Acceleration is the rate of change of velocity divided by time. Therefore, in indoor navigation, the acceleration is calculated using the following formula.

(40) $$a=\frac{v_{f}-v_{i}}{t}$$

(41) $$a=\frac{\Delta v}{t}$$

In Equations (40) and (41), the a represent the acceleration in $m/s_{2}$, $v_{f}$ is the final velocity, $v_{i}$ is the initial velocity of the object, *t* represent the time in second, and Δv is the change in velocity in m/s.

**Figure 13:**
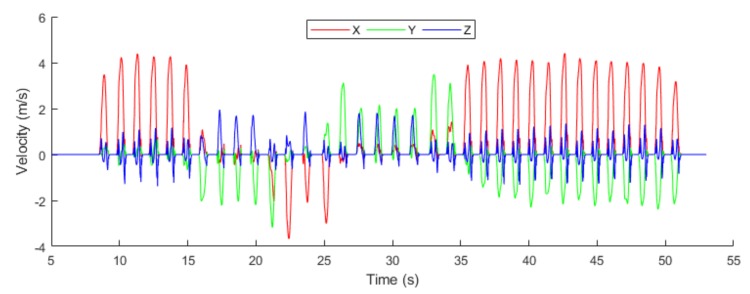
Velocity m/s.

In Figure 13, we calculate the 3-dimensional velocity of the object in an indoor environment. The velocity is used to measure how fast the object is moving; therefore in the proposed system, we calculate the velocity using the following formula.

(42) $$v=\frac{x_{f}-x_{i}}{t}$$

(43) $$v=\frac{\Delta x}{t}$$

In Equations (42) and (43), the *v* represent the velocity in m/s, $x_{f}$ is final position of the object, $x_{i}$ is the initial position, *t* is a time(*s*) in which change occur, and Δx represent the change in position.

**Figure 14:**
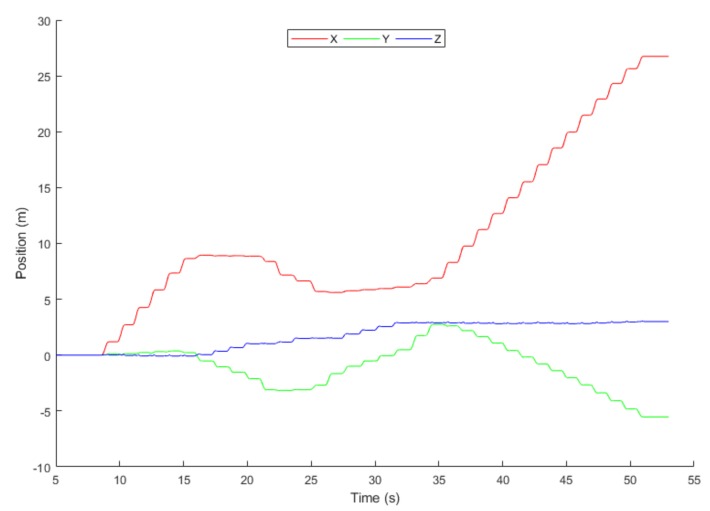
Position.

**Figure 15:**
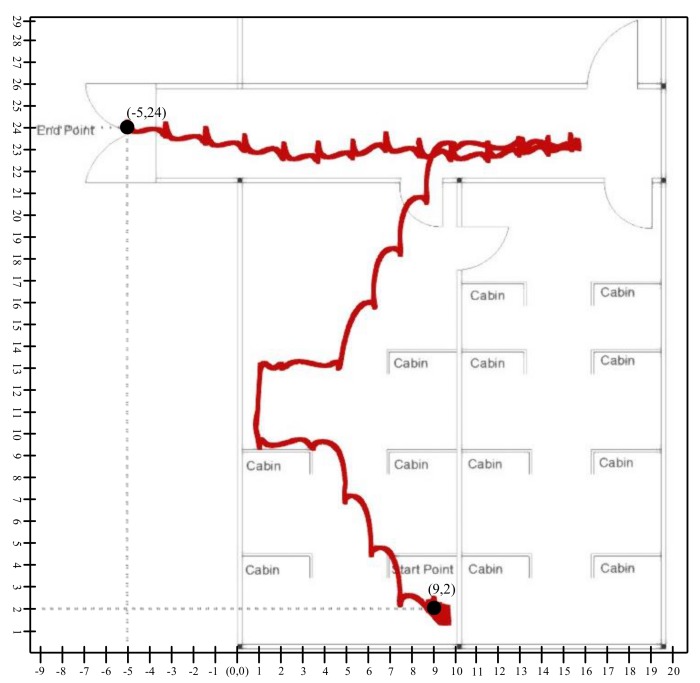
Person tracking scenario in Engineering building-4 of Jeju National University.

**Figure 16:**
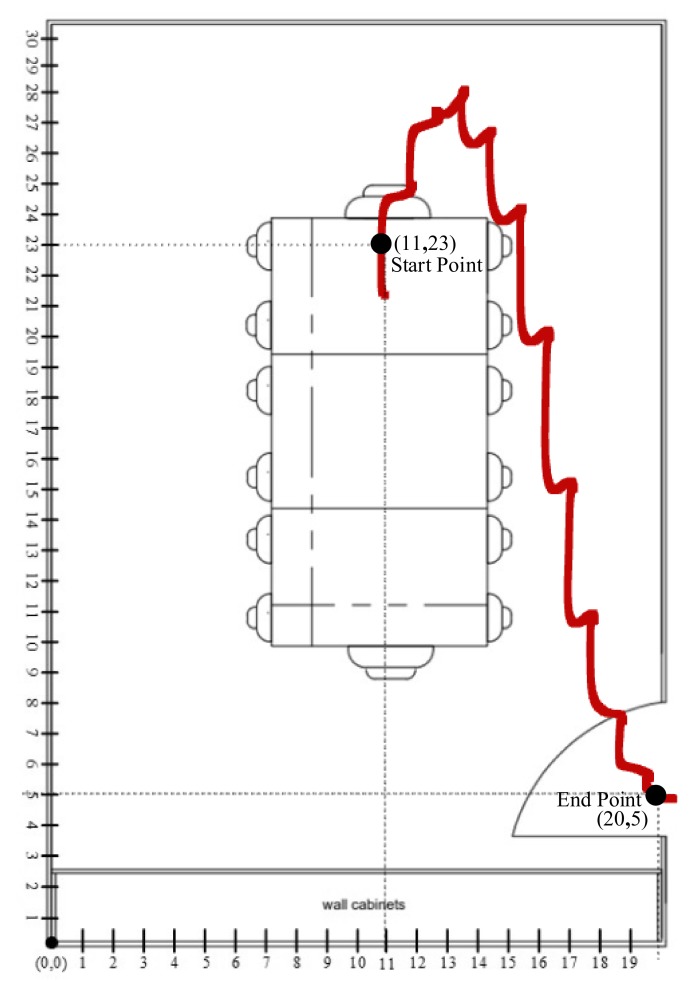
Person tracking scenario in ocean sciences building-5 of Jeju National University.

Figure 14 shows the position data plot of the 60 s of straight walk-in MCL Lab as a map in Figure 15 and Figure 16. We have concluded from this graph that drifts has significantly been reduced by the proposed system. However, there exists an error because the displacement on the axis should meet at the origin, but they didn’t. The *x*, *y*, and *z* represent the three-axis position.

Figure 15 and Figure 16 show the trajectory constrained in the X–Y plane from the top view. The Person starts walking from the start point and stops at the endpoint. As we saw in the graph, there is a drift in the start as it presents the person in stationary mode. In Figure 15, our lab has dimension of 20 × 29, where 12 is the length and 29 is the width. We have set our reference point on origin which is (0,0) therefore, our starting point is (9,2) with respect to reference point of the lab, which is (0,0). All the coordinates of the tracking line are referenced with respect to our reference point (0,0).

Similarly, in Figure 16, the conference has a dimension of 20 × 30, where 20 is the length and 30 is the width. We have set the reference point with respect to the origin, where starting point is (11,23) and the endpoint is (20,5).

**Figure 17:**
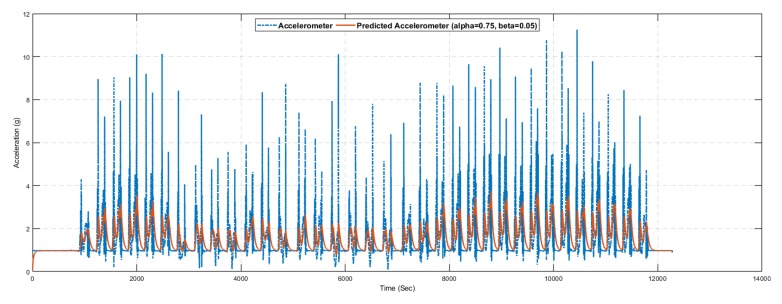
Accelerometer prediction results using the alpha–beta filter algorithm with selected values of alpha and beta.

**Figure 18:**
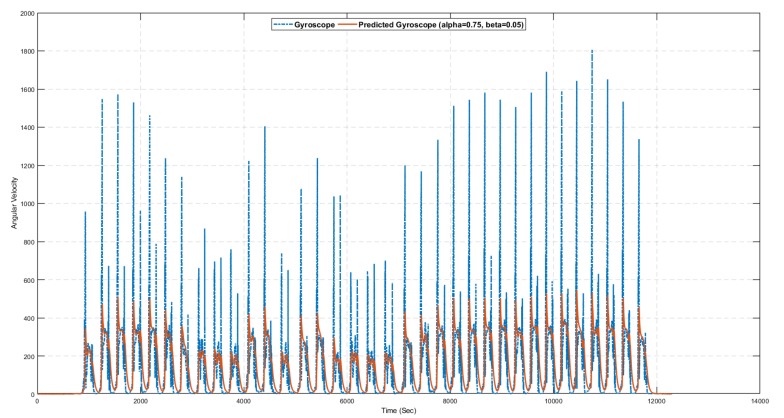
Gyroscope prediction results using the alpha–beta filter algorithm with selected values of alpha and beta.

Figure 17 and Figure 18 show the results of the alpha–beta filter with selected values of alpha and beta. The optimal values of alpha and beta are not fixed, and depend on the available dataset. It is challenging to choose the optimal values of alpha and beta in the alpha–beta filter manually. Therefore, multiple experiments were conducted with different values of alpha and beta; however, using alpha=0.75 and beta=0.05, we predict the required value of accelerometer and gyroscope for the noisy sensor reading.

Next, we present the results of the alpha–beta filter tuned with the proposed learning to prediction model. After tuning the ANN learning module, we used the trained model to improve the performance of the alpha–beta filter algorithm by appropriately tuning its parameters alpha and beta. In order to find the alpha-beta gain from the predicted error, we need to choose an appropriate value (i.e., *R*, the proportionality constant) called the error factor as represented in Equation (44).

(44) $$gain=\frac{error_{i}}{R}$$

**Figure 19:**
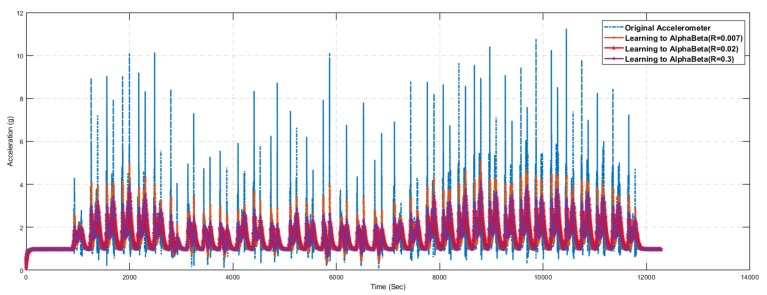
Accelerometer prediction results using the proposed learning to Alpha-Beta filter algorithm with selected error factor *R*.

**Figure 20:**
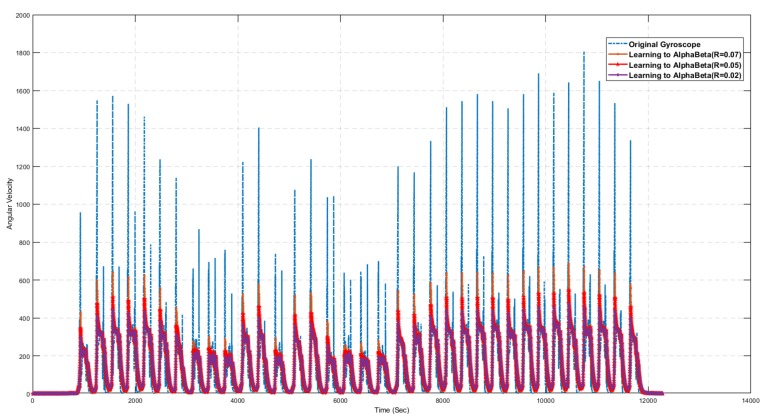
Gyroscope prediction results using the proposed learning to Alpha-Beta filter algorithm with selected error factor *R*.

Hence, experiments were conducted by varying the values of the error factor *R*. Figure 19 and Figure 20 show the prediction results of the alpha–beta filter algorithm using the learning module, varying the values of the error factor *R*. It is very difficult to comprehend the results presented in Figure 19 and Figure 20, as the difference between the results is not so obvious visually. Therefore, we used various statistical measures to summarize these results in the form of a single statical value for the quantifiable comparative analysis.

In Table 8, we computed the RMSE of the position with and without proposed learning to the prediction model. According to the results, we improved the accuracy of position estimation by 18%. The proposed learning to prediction model corrects the bias error by removing the noise and improving the accuracy of the system.

We have used three statistical measures that were used for performance comparison in terms of accuracy, i.e., Mean absolute deviation (MAD), Mean Squared Error (MSE), Root mean squared error (RMSE), and Mean absolute error (MAE). The formulas of these statistical measures are presented in Equations (45)–(47).

(45) $$MAD=\frac{\sum_{n}^{i=1}\left|Actual_{i}-\hat{Predicted_{i}}\right|}{n}$$

MAD is used to compute an average deviation found in the predicted values from the actual values. The calculation is done by dividing the sum of the absolute difference between the actual accelerometer and actual gyroscope $Actual_{i}$ and predicted accelerometer and gyroscope $\hat{Predicted_{i}}$ by the alpha–beta filter with the total number of the data items, i.e., *n*.

(46) $$MSE=\frac{\sum_{n}^{i=1}{\left(Actual_{i}-\hat{Predicted_{i}}\right)}^{2}}{n}$$

Similarly, MSE is considered the most widely used statistical measure in the performance evaluation of the prediction algorithms. Squaring the error magnitude not only removes the negative and positive error problems, but it also gives more penalty for higher misdirections as compared to low errors.

(47) $$RMSE=\sqrt{\frac{\sum_{n}^{i=1}{\left(Actual_{i}-\hat{Predicted_{i}}\right)}^{2}}{n}}$$

Finally, the mean absolute error (MAE) is the absolute error to measure the difference between two continuous variables.

(48) $$\frac{1}{n}\sum_{n}^{i=1}\left|\left(Actual_{i}-\hat{Predicted_{i}}\right)\right|$$

The problem with MSE is that it magnifies the actual error, which sometimes makes it difficult to realize and comprehend the actual error amount. This problem is resolved by the RMSE measure, which is obtained by simply taking the square root of MSE.

Table 9 presents the statistical summary of the results for the alpha–beta filter algorithm with and without a learning module. Comparative analysis shows that the alpha–beta filter with the proposed learning to prediction model results in an error factor R=0.02 (highlighted in bold), outperforming all other settings on all statistical measures. The best results for the alpha–beta filter without the learning module were obtained with alpha=0.75 and beta=0.05, which results in prediction accuracy of 2.49 in terms of RMSE. Similarly, the best results for the alpha–beta filter with the learning module were obtained with *R* = 0.02, which results in prediction accuracy of 2.38 in terms of RMSE. Figure 17, Figure 18, Figure 19 and Figure 20 show sample results for best cases of an alpha–beta filter with and without the ANN-based learning module. The relative improvement in prediction accuracy of the proposed learning to prediction model (best case), when compared to the best and worst-case result of the alpha–beta filter without the learning module, was 4.41% and 11.19% in terms of RMSE metric, respectively. Significant improvement in prediction accuracy gives us the confidence to further explore the application of the proposed learning to prediction model to improve the performance of other prediction algorithms.

## 6. Conclusions

In this article, we presented a novel learning to prediction model to improve the performance of prediction algorithms under dynamic conditions. The proposed model enabled conventional prediction algorithms to adapt to dynamic conditions through continuous monitoring of its performance and tuning of its internal parameters. To evaluate the effectiveness of the proposed learning to the prediction model, we developed an ANN-based learning module to improve the prediction accuracy of the alpha–beta filter algorithm as a case study. The proposed learning to prediction scheme improved the performance of the alpha–beta filter prediction by dynamically tuning its internal parameter α and β, i.e., estimated error in measurement. The ANN-based learning to prediction takes three input parameters (i.e., the current sensor reading (i.e., accelerometer and gyroscope) and alpha–beta predicted reading) in order to predict the estimated noise in sensor readings. Afterwards, the estimated error in the measurement parameter, i.e., α,β in the alpha–beta filter is updated by dividing the estimated error with a noise factor *R*.

## Figures and Tables

**Table 1:** Critical analysis of signal properties.

Signal	Property	Measurement	Metric
Angle of Arrival (AOA)	Angle-based	High accuracy at room level	Complex, expensive and low accuracy at wide coverage
Received Signal Strength Indication (RSSI)	Signal-based (RSS)	Medium accuracy	Low cost
Time of Arrival (TOA)	Distance-based	High accuracy	Complex and expensive
Time Difference of Arrival (TDOA)	Distance-based	High accuracy	Expensive
Hop-Based	Signal-based	High accuracy	Complex and expensive with short range coverage
Interferometry	Signal-based	Medium accuracy	Complex with low accuracy
Return Time of Flight (RToF)	Signal-based	Low accuracy	Short range coverage

**Table 2:** Critical analysis of positioning algorithms.

Positioning Algorithm	Signal Property	Pros	Cons
Triangulation	AOA	High accuracy at room level	Complex, expensive and low accuracy at wide coverage
Trilateration	TOA/TDOA	Medium accuracy	Complex and expensive
Proximity	RSSI	High accuracy	Complex and expensive
Connectivity/ Neighbourhood	RSSI/ Hop-based	High accuracy	Complex, expensive, short coverage
Scene analysis/fingerprinting	RSSI	High performance	Complex, expensive, medium accuracy and time consuming

**Table 3:** Critical analysis of positioning technologies.

Technology	Technique	Algorithm	Accuracy	Cost	Complexity	Scalability	Real-time
Infrared	Trilateration	TOA, TDOA	Medium	Low	High	Medium	Yes
Audible sound	Trilateration	TOA	Medium	Medium	Medium	Medium	Yes
Magnetic	Triangulation	AOA, TOA	High	High	High	Low	Yes
Bluetooth	Trilateration, fingerprinting	TDOA, RSSI	Low	Medium	Medium	Medium	Yes
WLAN	Trilateration, fingerprinting	TDOA, RSSI	Low	Medium	High	Medium	Yes
RFID	Fingerprinting	RSSI	Low	Medium	Medium	High	Yes
UWB	Trilateration	TOA, TDOA	High	Medium	Medium	Medium	Yes
NFC	Proximity	RSSI	High	Low	Low	High	No
WSN	Fingerprinting	RSSI	Medium	Medium	Medium	Medium	Yes
PDR/INS	DR	EKF, PF	Medium	Low	Low	Medium	Yes

**Table 4:** Characteristic of NGIMU.

Sensor	Description	
Gyroscope	Range	±$2000^{\circ }$/s
	Resolution	$0.06^{\circ }$/s
	Sample Rate	400 Hz
Accelerometer	Range	±16 g
	Resolution	490μg
	Sample Rate	400 Hz
Magnetometer	Range	±1300μT
	Resolution	∼0.3 μT
	Sample Rate	∼20 Hz

**Table 5:** Development environment for the proposed inertial tracking in indoor navigation.

Component	Description
IDE	MATLAB R2018a
Operating System	Window 10
CPU	Intel(R) Core(TM) i5-8500 CPU@3.00GHz
Memory	8GB
Signal Processing Filter	Butterworth Digital Filter
Data Smoothing Algorithm	Alpha-Beta filter

**Table 6:** Development environment for the proposed Learning to Prediction Model.

Component	Description
IDE	MATLAB R2018a
Operating System	Window 10
CPU	Intel(R) Core(TM) i5-8500 CPU@3.00GHz
Memory	8GB
Artificial Neural Network	Feed Forward Backpropagation
Neuron in Hidden Layer	10
Neuron in output Layer	2
Number of Input	3
Prediction Algorithm	Alpha-Beta filter

**Table 7:** Prediction accuracy in terms of RMSE for training and testing dataset using four-fold cross validation in indoor navigation system.

Experiment ID	ANN Configuration			Model 1		Model 2		Model 3		Model 4		Model Average (Test Cases)	Experiments Average (Test Case)
	Activation Function	Hidden Layers	Learning Rate	Trainina	Test	Trainina	Test	Trainina	Test	Trainina	Test		
1	Sigmoid	10	0.1	0.25	0.22	0.12	0.12	0.25	0.25	0.21	0.24	0.20	0.23
2	Sigmoid	10	0.1	0.26	0.23	0.25	0.26	0.28	0.28	0.50	0.35	0.28	
3	Sigmoid	10	0.1	0.29	0.24	0.15	0.17	0.10	0.12	0.32	0.35	0.22	
1	Linear	10	0.1	4.56	5.22	5.06	3.20	4.48	5.07	4.58	4.90	4.59	4.59
2	Linear	10	0.1	4.56	5.22	5.06	3.20	4.48	5.07	4.58	4.90	4.59	
3	Linear	10	0.1	4.56	5.22	5.06	3.20	4.48	5.07	4.58	4.90	4.59	
**1**	**Sigmoid**	**10**	**0.2**	**0.19**	**0.15**	**0.08**	**0.09**	**0.20**	**0.22**	**0.24**	**0.27**	**0.18**	**0.19**
**2**	**Sigmoid**	**10**	**0.2**	**0.24**	**0.25**	**0.18**	**0.20**	**0.19**	**0.20**	**0.18**	**0.19**	**0.21**	
**3**	**Sigmoid**	**10**	**0.2**	**0.24**	**0.19**	**0.15**	**0.16**	**0.24**	**0.24**	**0.22**	**0.23**	**0.20**	
1	Linear	10	0.2	4.44	5.18	5.03	3.17	4.49	5.04	4.55	4.87	4.56	4.56
2	Linear	10	0.2	4.44	5.18	5.03	3.17	4.49	5.04	4.55	4.87	4.56	
3	Linear	10	0.2	4.44	5.18	5.03	3.17	4.49	5.04	4.55	4.87	4.56	
1	Sigmoid	15	0.1	1.15	0.91	0.27	0.34	0.34	0.33	0.24	0.27	0.46	0.33
2	Sigmoid	15	0.1	0.13	0.11	0.23	0.25	0.23	0.20	0.31	0.31	0.21	
3	Sigmoid	15	0.1	0.57	0.45	0.34	0.36	0.22	0.22	0.19	0.23	0.31	
1	Linear	15	0.1	4.45	5.19	5.04	3.18	4.50	5.05	4.56	4.88	4.57	4.57
2	Linear	15	0.1	4.45	5.19	5.04	3.18	4.50	5.05	4.56	4.88	4.57	
3	Linear	15	0.1	4.45	5.19	5.04	3.18	4.50	5.05	4.56	4.88	4.57	
1	Sigmoid	15	0.2	0.27	0.23	0.56	0.91	0.19	0.22	0.40	0.40	0.44	0.30
2	Sigmoid	15	0.2	0.24	0.20	0.22	0.25	0.26	0.29	0.20	0.23	0.24	
3	Sigmoid	15	0.2	0.25	0.19	0.20	0.24	0.21	0.22	0.21	0.24	0.22	
1	Linear	15	0.2	4.45	5.19	5.04	3.18	4.50	5.05	4.56	4.88	4.57	4.57
2	Linear	15	0.2	4.45	5.19	5.04	3.18	4.50	5.05	4.56	4.88	4.57	
3	Linear	15	0.2	4.45	5.19	5.04	3.18	4.50	5.05	4.56	4.88	4.57	

**Table 8:** Estimating error in position with and with proposed learning to prediction model.

Experiment ID	Position Error	Position Error with Proposed Learning to Prediction Model
1	0.130 mm	0.102 mm
2	0.115 mm	0.098 mm
3	0.135 mm	0.112 mm

**Table 9:** Statistical summary of the alpha–beta filter prediction results with and without the ANN-based learning module.

Metric	Alpha-Beta Filter		Alpha-Beta with Learning Module	
	α=0.75,β=0.05	α=1.45,β=0.75	*R* = 0.02	*R* = 0.1
RMSE	**2.494**	2.527	**2.388**	2.481
MAD	**0.163**	0.166	**0.156**	0.165
MSE	**6.222**	6.388	**5.701**	6.155
MAE	**0.997**	1.137	**0.931**	1.2156
